# Primary Lacrimal Sac Diffuse Large B-cell Lymphoma Treated With Local Radiotherapy Alone: A Case With No Relapse After 21 Years of Follow-Up

**DOI:** 10.7759/cureus.95411

**Published:** 2025-10-25

**Authors:** Toshihiko Matsuo, Takehiro Tanaka, Mitsuhiro Takemoto

**Affiliations:** 1 Healthcare Science, Graduate School of Interdisciplinary Science and Engineering in Health Systems, Okayama University, Okayama, JPN; 2 Ophthalmology, Okayama University Hospital, Okayama, JPN; 3 Pathology, Graduate School of Medicine, Dentistry, and Pharmaceutical Sciences, Okayama University, Okayama, JPN; 4 Radiotherapy, Himeji Red Cross Hospital, Himeji, JPN

**Keywords:** diffuse large b-cell lymphoma, excisional biopsy, lacrimal sac, laser photocoagulation, macular edema, pathology, radiation cataract, radiation retinopathy, radiotherapy, vitrectomy

## Abstract

Primary lacrimal sac lymphoma is rare and diagnosed as diffuse large B-cell lymphoma in a predominant histopathological type. Systemic chemotherapy would be the standard of care, but local radiotherapy may be a treatment option toward a localized lesion. The present patient is a 54-year-old otherwise healthy woman with a right lacrimal sac mass, which was proven by excisional biopsy to be diffuse large B-cell lymphoma. Since she did not have any other systemic lesions on gallium scintigraphy and neck-to-abdominal computed tomography scans, which were the standard procedure at that time, she underwent local radiotherapy at 40 Gy. Two years later, at the age of 56 years, she developed radiation retinopathy with macular edema in the right eye and had spotty laser photocoagulation in the nasal half of the fundus. At the age of 57 years, she developed radiation cataract and underwent cataract surgery with intraocular lens implantation in the right eye. At the age of 58 years, the macular edema in the right eye became worse and remained active, resulting in poor visual acuity of 0.1. She thus underwent 25-gauge vitrectomy in the right eye to peel off the adhering posterior vitreous surface, together with the internal limiting membrane, as the standard procedure at that time. The visual acuity in the right eye was elevated to 0.6. She maintained the visual acuity afterward and had no relapse of lymphoma in 21 years from the diagnosis of primary right lacrimal sac diffuse large B-cell lymphoma. Local radiotherapy would still be a treatment option for localized lymphoma lesions such as primary lacrimal sac lymphoma.

## Introduction

The lacrimal sac is the part of the lacrimal drainage system which conveys ocular surface fluid from the lacrimal puncta, canaliculi, sac, and nasolacrimal duct to the nasal cavity. Lacrimal sac tumors should be differentiated in the list of dacryocystitis as chronic inflammation, and benign and malignant tumors such as adenocarcinoma of lacrimal sac epithelial origin, malignant melanoma, and lymphoproliferative diseases such as benign lymphoid hyperplasia and lymphoma [1]. From a different angle, the lacrimal sac is part of the ocular adnexa, which supports the eyeball and is often involved in lymphoproliferative diseases [2-5].

Lymphoma is the uncontrolled proliferation of a certain lineage of lymphoid cells and is classified into different histopathological types, based on the supposed cell of origin. Among different types of lymphoma, diffuse large B-cell lymphoma is considered as aggressive, based on a high speed of proliferation and a high rate of progressive invasion to other areas of the body. According to previous reviews [6-9], primary lacrimal sac lymphoma is predominantly classified into the pathological entity of diffuse large B-cell lymphoma. As a treatment option for primary lacrimal sac diffuse large B-cell lymphoma, systemic chemotherapy was chosen in most previous case reports [9-20], while the combination of chemotherapy and radiotherapy was used in a few reports [21-23]. Local radiotherapy only was described in the limited number of cases with primary lacrimal sac diffuse large B-cell lymphoma [24-27]. In the case of a localized lymphoma lesion such as primary lacrimal sac lymphoma, local radiotherapy remains a treatment option [28,29], on the general understanding that systemic chemotherapy is the standard of care. In this study, we report a patient with primary lacrimal sac diffuse large B-cell lymphoma who underwent local radiotherapy only and showed no relapse in the follow-up of 21 years.

## Case presentation

**Figure 1:**
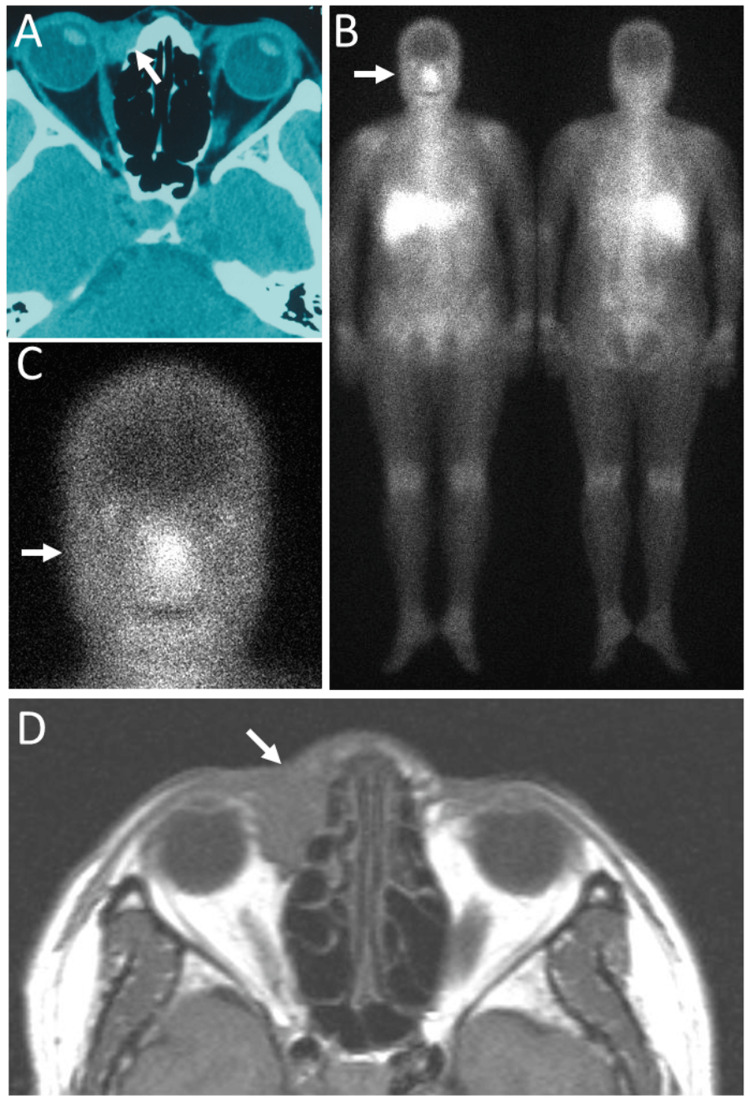
Computed tomography scan, gallium scintigraphy, and magnetic resonance imaging at age 54 years Computed tomography scan (A), showing right lacrimal sac mass (arrow) at age 54 years. After excisional biopsy to confirm diffuse large B-cell lymphoma, gallium scintigraphy (B, C), showing abnormal high uptake only at the right lacrimal sac area (arrows). T1-weighted magnetic resonance imaging (D), showing homogeneous right lacrimal sac mass (arrow).

**Figure 2:**
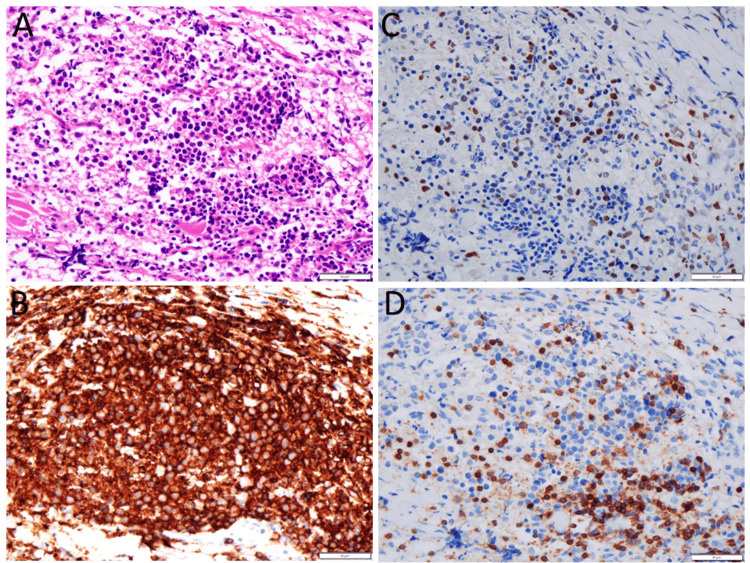
Pathology images at age 54 years Excisional biopsy of right lacrimal sac mass at age 54 years, showing diffuse infiltration with anomalous large cells in hematoxylin-eosin stain (A), positive for CD20 (B) and Ki-67 (C), admixed with a smaller number of CD3-positive T cells (D). The pathological diagnosis is diffuse large B-cell lymphoma. Scale bar = 50 µm.

A 54-year-old woman experienced epiphora in the right eye and noticed a painless mass at the medial canthus of the right eye, which had gradually increased over half a year. At referral, a well-defined movable elastic-hard mass in the long diameter of 2 cm was localized to the right lacrimal sac area with no skin adhesion. She did not have fever, weight loss, or night sweats. The visual acuity was 1.5 in both eyes. The slit-lamp and fundus examinations were all normal. The physical examinations were also normal and did not detect lymphadenopathy. Blood examinations, including complete blood cell counts and blood chemistry as well as urinalysis, were all normal. In past history, she had appendicitis at the age of eight years. She had been taking a β-blocker drug for hypertension and tachycardia. Computed tomography scans at referral showed a lacrimal sac mass on the right side (Figure 1A). Excisional biopsy of the lacrimal sac mass proved pathologically diffuse large B-cell lymphoma (Figure 2A): CD20-positive large B cells (Figure 2B), which were highly positive for Ki-67 (Figure 2C), admixed with a smaller number of CD3-positive T cells (Figure 2D). The lymphoid cells were negative for CD10. As fluorodeoxyglucose positron emission tomography was not available, gallium scintigraphy was performed as the standard procedure at that time to show a high uptake site only in the right lacrimal sac lesion and no other abnormal uptake site systemically (Figures 1B, 1C). Cervical, chest-to-abdominal computed tomography scans disclosed neither lymphadenopathy nor mass lesion. Magnetic resonance imaging confirmed the right lacrimal sac mass in an internal homogeneous pattern (Figure 1D).

**Figure 3:**
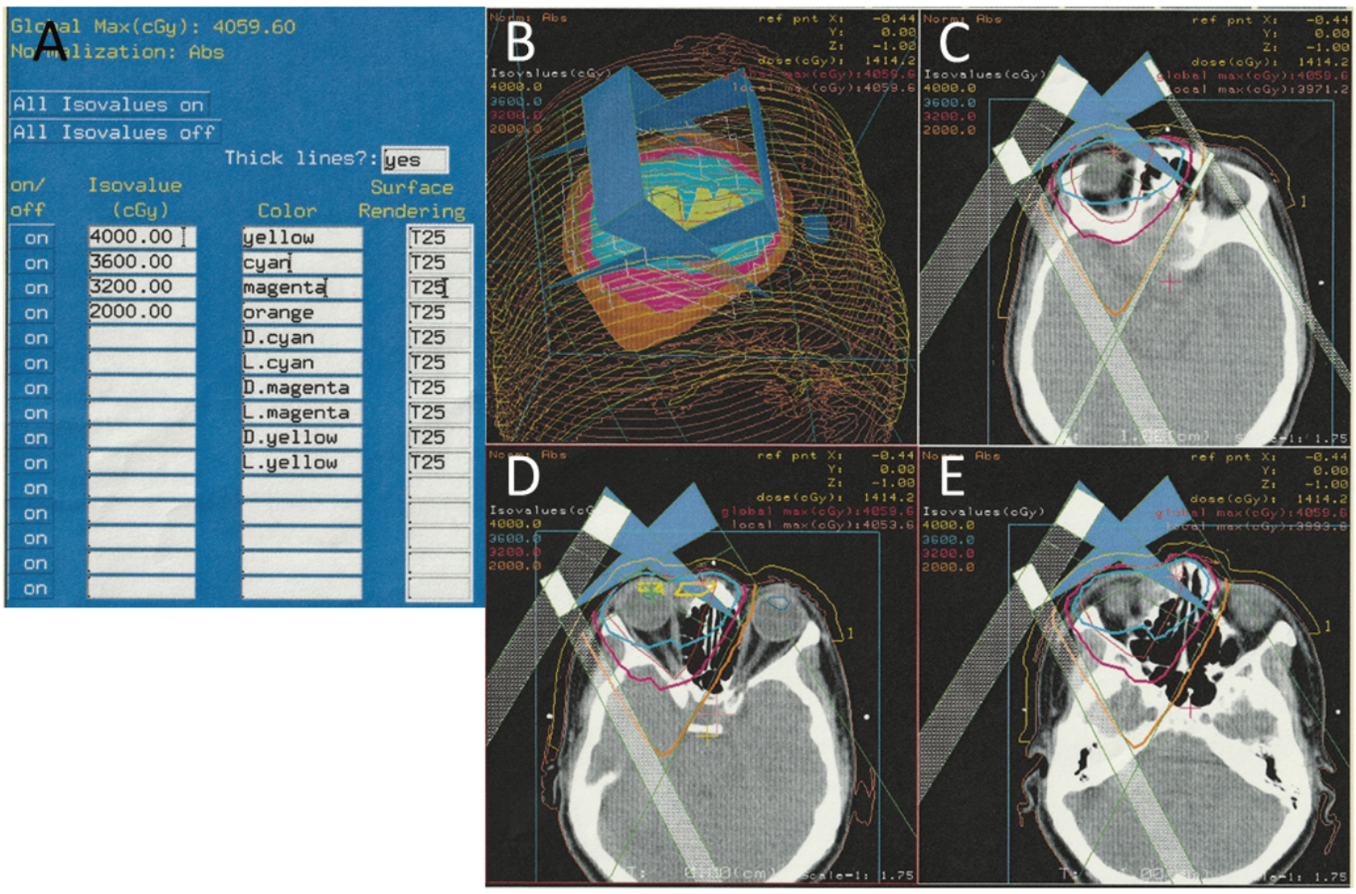
Radiotherapy plan at age 54 years Local radiotherapy plan for primary right lacrimal sac diffuse large B-cell lymphoma at age 54 years. Radiation dose distribution (A) with different color lines (maximum dose at 40 Gy). Front view (B) and different axial sections of computed tomography scans (C, D, E).

**Figure 4:**
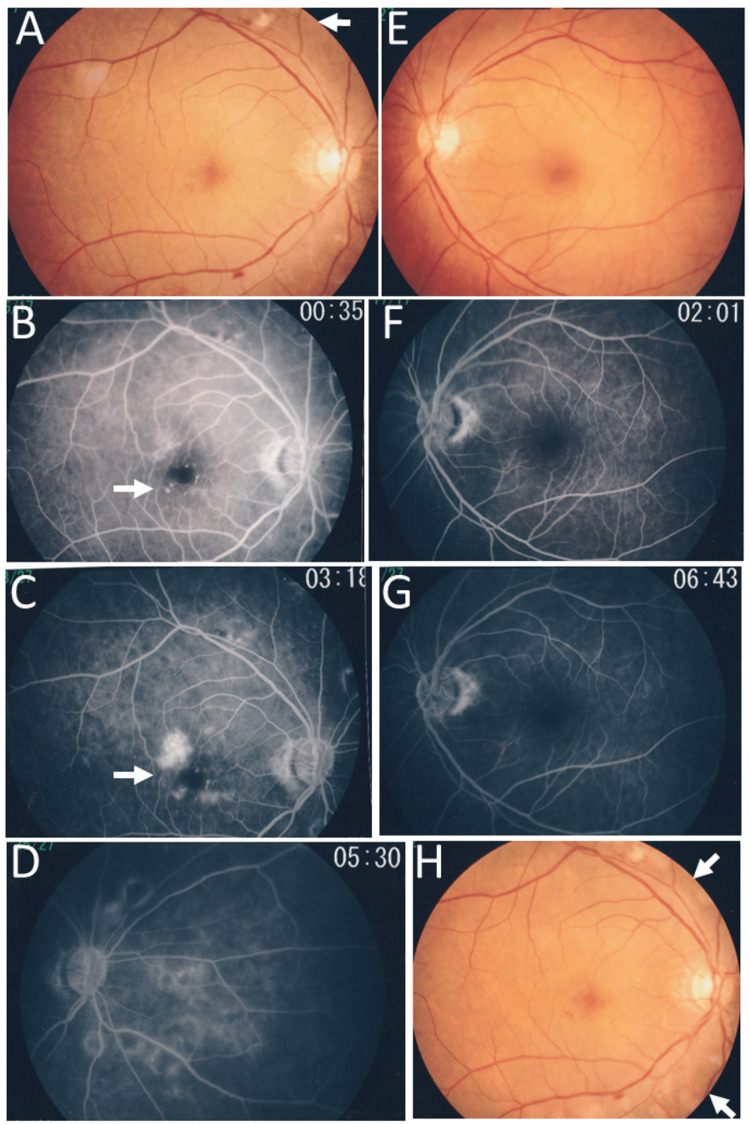
Fundus photographs and fluorescein angiograms at age 56 years Fundus photographs (right eye: A, left eye: E), showing cotton-wool spots (arrow, A) and macular edema in right eye. Fluorescein angiography, showing perifoveal microaneurysms in early phase (arrow, B) with late-phase leakage (arrow, C) as well as wide avascular areas with capillary leakage on the nasal side of the fundus (D) in the right eye. Fluorescein angiography in left eye is normal in early (F) and late (G) phases. Spotty laser photocoagulation (H) outside the superior and inferior vascular arcades (arrows) and on nasal side one week later.

In the consultation with a hematologist and a radiation oncologist, she was recommended local radiotherapy and underwent 40 Gy radiation (2 Gy each in 20 fractions), based on a design of two anterior oblique fields (Figures 3A-3E). She was stable with no symptoms in two years until the age of 56 years when she noticed blurring and metamorphopsia in the right eye. She maintained the best-corrected visual acuity of 1.2 in both eyes. Fundus examinations revealed macular edema and cotton-wool spots in the right eye (Figure 4A), and fluorescein angiography disclosed perifoveal microaneurysms (Figure 4B) with late-phase leakage (Figure 4C) as well as wide avascular areas with capillary leakage (Figure 4D) on the nasal half of the fundus in the right eye. The left eye was normal (Figures 4E-4G). Spotty laser photocoagulation was applied to the nasal side, the upper and lower part of the fundus outside the superior and inferior vascular arcades (Figure 4H).

**Figure 5:**
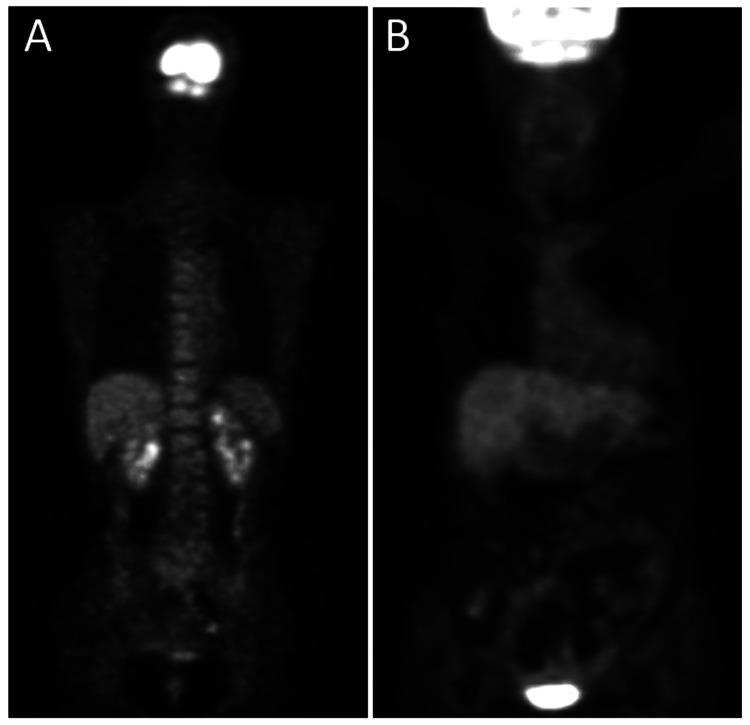
Positron emission tomography at ages 57 and 68 years Fluorodeoxyglucose positron emission tomography at ages 57 years (A) and 68 years (B), showing no abnormal uptake site.

**Figure 6:**
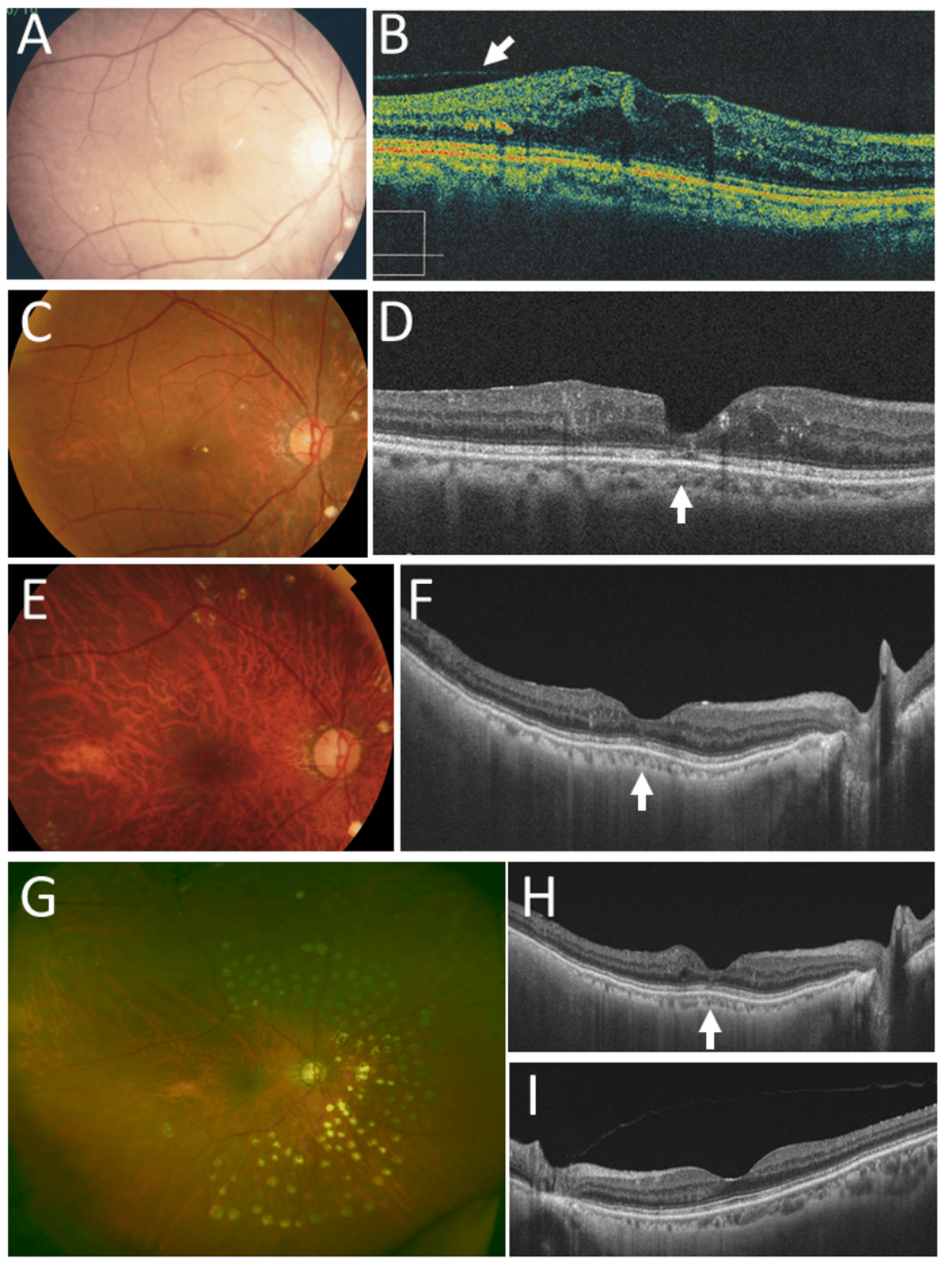
Fundus photographs and optical coherence tomography at ages 58, 60, 67, and 68 years Fundus photograph (A) and horizontal section image of optical coherence tomography (B) in right eye at age 58 years, 3 weeks before vitrectomy, showing macular edema with posterior vitreous surface adhesion (arrow, B). Fundus photographs and horizontal section images of optical coherence tomography at ages 60 years (C, D) and 67 years (E, F), showing no macular edema (arrows, D, F) in right eye. Wide-field fundus photograph (G) and horizontal section images of optical coherence tomography in right eye (H) and left eye (I) at age 68 years, showing spotty laser photocoagulation scars in nasal-half fundus and no macular edema (arrow, H).

One year later, at the age of 57 years, fluorodeoxyglucose positron emission tomography which became available showed no abnormal uptake systemically (Figure 5A). Half a year later, she underwent cataract surgery with intraocular lens implantation for radiation cataract in the right eye. In the next half a year later, at the age of 58 years, she still showed macular edema in the right eye (Figure 6A) and the best-corrected visual acuity in the right eye dropped to 0.1. Optical coherence tomography revealed macular edema with posterior vitreous adhesion (Figure 6B). The patient underwent 25-gauge vitrectomy to peel off the posterior vitreous surface and internal limiting membrane with forceps, as the standard procedure at that time. One month later, the macular edema subsided and the visual acuity in the right eye was elevated to 0.2.

Two years later, at the age of 60 years, she maintained the absence of macular edema (Figures 6C, 6D) in the right eye with the visual acuity of 0.5. At the age of 67 years, the macular structure in the right eye was normal with the somewhat disrupted photoreceptor ellipsoid zone at the foveal center (Figures 6E, 6F). At the age of 68 years, she maintained the visual acuity of 0.6 and had no macular edema in the right eye with laser photocoagulation scars in the nasal half of the fundus (Figure 6G). Optical coherence tomography showed minimal disruption of the photoreceptor ellipsoid zone in the right eye (Figure 6H), compared with the normal ellipsoid zone in the left eye (Figure 6I). At this time, fluorodeoxyglucose positron emission tomography showed no abnormal uptake systemically (Figure 5B). At the age of 75 years, she was healthy and used topical 0.005% latanoprost and combination solution of 1% dorzolamide and 0.5% timolol as intraocular pressure-lowering eye drops for open-angle glaucoma in both eyes. The best-corrected visual acuity was 0.6 in the right eye with intraocular lens implantation and 1.5 in the left eye with no cataract. The intraocular pressure was 13 mmHg in both eyes.

## Discussion

The present patient is a 54-year-old otherwise healthy woman with a right lacrimal sac mass, which was proven by excisional biopsy to be diffuse large B-cell lymphoma. Since she did not have any other systemic lesion in gallium scintigraphy and neck-to-abdominal computed tomography scans, which were the standard procedure at that time, she underwent local radiotherapy at 40 Gy. Two years later, at the age of 56 years, she developed radiation retinopathy with macular edema in the right eye and had spotty laser photocoagulation in the nasal half of the fundus. At the age of 57 years, she developed radiation cataract and underwent cataract surgery with intraocular lens implantation in the right eye. At the age of 58 years, the macular edema in the right eye became worse and remained active, resulting in poor visual acuity of 0.1. She thus underwent 25-gauge vitrectomy in the right eye to peel off the adhering posterior vitreous surface, together with the internal limiting membrane, as the standard procedure at that time. The visual acuity in the right eye was elevated to 0.6. She maintained the visual acuity afterward and had no relapse of lymphoma in 21 years from the diagnosis of primary right lacrimal sac diffuse large B-cell lymphoma.

Table 1 summarizes five patients, including the present patient, with primary lacrimal sac diffuse large B-cell lymphoma who were treated only with local radiotherapy [24-27]. The patients were three females and two males with the age at the presentation ranging from 10 to 78 years at a median of 63 years. The lacrimal sac lymphoma was on the right side in two patients and on the left side in three patients. Each patient noticed a painless mass with epiphora as symptoms at the presentation. All patients underwent incisional or excisional biopsy to reach the pathological diagnosis of diffuse large B-cell lymphoma, except for one (Case 4) who underwent dacryocystectomy after an intraoperative frozen-section diagnosis of lymphoma in the planned surgery of dacryocystorhinostomy. The dose of local radiotherapy ranged from 30.6 Gy to 45 Gy in four patients, including the present patient, while the dose was not described in one patient (Case 2). The follow-up periods in four patients of the previous case reports ranged from one year to three years with no relapse, in contrast with 21 years with no relapse in the present patient (Case 5). No complication was described in the previous four cases with the short follow-up period, except for one child (Case 1) and one adult (Case 2) who underwent dacryocystorhinostomy for nasolacrimal duct stenosis, five months and 18 months later, respectively. The reason for the choice of local radiotherapy was described only in one patient (Case 3), stating that chemotherapy was not done because of poor performance status at the old age.

**Table 1:** Clinical features of five patients with primary lacrimal sac diffuse large B-cell lymphoma treated only by local radiotherapy, including the present patient CT, computed tomography scan; MRI, magnetic resonance imaging; PET, positron emission tomography.

Case No./Gender	Age at first visit	Laterality of lacrimal sac mass	Symptoms	Imaging of lacrimal sac mass	Biopsy or surgery	Staging	Local radiotherapy	Relapse and complications	Follow-up years	Authors (year)
1/Male	10 years	Left	1-year-long epiphora, 2-month-long painless swelling	Not described	Incisional biopsy	Negative bone marrow biopsy	36 Gy	No relapse, Dacryocystorhinostomy for nasolacrimal duct stenosis 5 months later	1 year	Carlin and Henderson (1974) [24]
2/Female	63 years	Left	1-month-long epiphora and painless swelling	Heterogeneous mass by CT	Excisional biopsy	No other lesion by CT, Negative bone marrow biopsy	Dose not described	No relapse, Dacryocystorhinostomy for nasolacrimal duct stenosis 18 months later	2.5 years	Jordan and Nerad (1988) [25]
3/Female	78 years	Left	6-month-long epiphora, 2-month-long painless swelling	Heterogeneous soft tissue mass, causing exophthalmos by CT	Incisional biopsy	No other lesion by CT, Negative bone marrow biopsy	45 Gy (Chemotherapy not done because of poor performance status at old age)	No relapse, No complication	3 years	Venkitaraman and George (2007) [26]
4/Male	77 years	Right	3-year-long epiphora, 6-month-long painless swelling	Homogeneous mass by MRI	Dacryocystectomy	No abnormal uptake in PET	30.6 Gy	No relapse, No complication	1 year	Kajita et al. (2010) [27]
5/Female	54 years	Right	6-month-long epiphora and painless swelling	Homogeneous mass by CT and MRI	Excisional biopsy	No other abnormal uptake in gallium scintigraphy, No abnormal uptake in follow-up PET 3 years and 14 years later	40 Gy	No relapse, Laser photocoagulation for radiation retinopathy and maculopathy 2 years later, Cataract surgery for radiation cataract 3 years later, 25-gauge vitrectomy for radiation maculopathy 4 years later	21 years	This case

In the field of ophthalmology, ocular adnexal lymphoma [2-5] and primary intraocular lymphoma [30-33] are two major entities of lymphoma. Basically, the treatment strategies are determined by histopathological types of lymphoma. It is indeed difficult to choose a better treatment option in the case of a localized lymphoma lesion. In previous cases of primary lacrimal sac diffuse large B-cell lymphoma, systemic chemotherapy was the prevailing treatment, while local radiotherapy only was a rare choice of treatment. The present patient is unique at the point that she showed no relapse in 21 years of follow-up. In contrast with short follow-up periods in the four previous patients who underwent local radiotherapy for primary lacrimal sac diffuse large B-cell lymphoma [24-27], the long-term follow-up might be the reason why the present patient was detected to have radiation retinopathy and radiation cataract, two years and three years, respectively, after the local radiotherapy.

Radiation retinopathy, especially radiation maculopathy, is a major complication of local radiotherapy [34,35]. It should be noted that the nasal half of the fundus in the right eye showed retinal capillary occlusion and leakage. The nasal half of the right eyeball, including the macular area, was indeed the area of irradiation at local radiotherapy toward the right lacrimal sac lymphoma. Capillary obliteration outside the posterior pole, usually outside the superior and inferior vascular arcades, can be photocoagulated by laser to stabilize the vascular leakage. In contrast, it is difficult to manage macular edema caused by perifoveal leakage which is derived from microaneurysms and capillary insufficiency. Recently, anti-vascular endothelial growth factor agents are available as a choice of treatment for radiation-induced maculopathy [34,35]. In the present patient who showed persistent macular edema two years after laser photocoagulation, 25-gauge vitrectomy to peel off the posterior vitreous surface and internal limiting membrane was chosen as the standard in the year 2008.

## Conclusions

Systemic chemotherapy is the standard of care in cases with a pathological diagnosis of diffuse large B-cell lymphoma. Even in this general understanding, local radiotherapy would be a treatment option for a localized lesion of diffuse large B-cell lymphoma. The present patient with primary lacrimal sac diffuse large B-cell lymphoma is an example of no relapse in the long term, only with local radiotherapy. Radiation retinopathy and radiation cataract in the patient could be managed successfully with laser treatment and eye surgeries.

## References

[1] Matsuo T, Tanaka T, Yamasaki O (2019). Lacrimal sac malignant melanoma in 15 Japanese patients: case report and literature review. J Investig Med High Impact Case Rep.

[2] Matsuo T, Ichimura K, Okada H (2010). Clonal analysis of bilateral, recurrent, or systemically multifocal ocular adnexal lymphoma. J Clin Exp Hematop.

[3] Matsuo T, Tanaka T, Okada K, Notohara K, Fujii K, Fujii N (2023). Bilateral lacrimal gland mantle cell lymphoma in 11-year follow-up: case report and review of 48 cases with ocular adnexal presentation in the literature. J Investig Med High Impact Case Rep.

[4] Matsuo T, Tanaka T (2024). Spontaneous regression and rare relapse after excisional biopsy in long-term observation of 31 patients with primary conjunctival lymphoma. J Clin Exp Hematop.

[5] Matsuo T, Tanaka T, Fujii N (2025). Six-year remission with no relapse after four-time weekly rituximab only for bilateral ocular adnexal follicular lymphoma. Cureus.

[6] Sjö LD, Ralfkiaer E, Juhl BR (2006). Primary lymphoma of the lacrimal sac: an EORTC ophthalmic oncology task force study. Br J Ophthalmol.

[7] Singh S, Ali MJ (2020). Lymphoproliferative tumors involving the lacrimal drainage system: a major review. Orbit.

[8] Athanasopoulos M, Nomikos G, Samara P, Mastronikolis S, Tsilivigkos C, Mastronikolis NS (2024). Non‑Hodgkin's lymphomas of the lacrimal sac: current insights and future directions (Review). Med Int (Lond).

[9] Gao HW, Lee HS, Lin YS, Sheu LF (2005). Primary lymphoma of nasolacrimal drainage system: a case report and literature review. Am J Otolaryngol.

[10] Thomas A, Kong J, Eisenberg R (2010). Primary diffuse large B-cell lymphoma of the lacrimal sac. Otolaryngol Head Neck Surg.

[11] Palamar M, Midilli R, Ozsan N, Egrilmez S, Sahin F, Yagci A (2011). Primary diffuse large B-cell lymphoma of the lacrimal sac simulating chronic dacryocystitis. Auris Nasus Larynx.

[12] Tsao WS, Huang TL, Hsu YH, Chen N, Tsai RK (2016). Primary diffuse large B cell lymphoma of the lacrimal sac. Taiwan J Ophthalmol.

[13] Zarrabi K, Desai V, Yim B, Gabig TG (2016). Primary diffuse large B-cell lymphoma localized to the lacrimal sac: a case presentation and review of the literature. Case Rep Hematol.

[14] Marunaka H, Orita Y, Tachibana T (2018). Diffuse large B-cell lymphoma of the lacrimal sac arising from a patient with IgG4-related disease. Mod Rheumatol.

[15] Robinette J, White C (2019). An unusual presentation of large B-cell lymphoma. Cureus.

[16] Parikh D, Rodgers R, Kodsi S (2019). Primary lacrimal sac diffuse large B-cell lymphoma in a child. J AAPOS.

[17] Kakutani S, Takahashi Y, Valencia MR, Kakizaki H (2018). Diffuse large B-cell lymphoma of the lacrimal sac in a Japanese patient. Case Rep Ophthalmol.

[18] Kim J, Kim J, Baek S (2022). Diffuse large B-cell lymphoma arising in the lacrimal sac. J Craniofac Surg.

[19] Soman S, Thambi SM, Jacob PM (2022). Lacrimal sac diffuse large B-cell lymphoma: a case report. Indian J Ophthalmol.

[20] Sultan S, Ibrahim T, Habib A, Hussein F (2025). Complete response of unilateral primary lacrimal sac diffuse large B-cell lymphoma treated without radiotherapy in an adult: a rare case report and review of the literature. Cancer Treat Res Commun.

[21] Nakamura K, Uehara S, Omagari J (1997). Primary non-Hodgkin's lymphoma of the lacrimal sac: a case report and a review of the literature. Cancer.

[22] Sabundayo MS, Takahashi Y, Kakizaki H (2019). Lacrimal sac lymphoma: a series of Japanese patients. Eur J Ophthalmol.

[23] Malzone MG, Di Meglio M, Furgiuele D, Galantuomo N, Alfano S, Mossetti G (2021). Primary non-Hodgkin diffuse large B-cell lymphoma of the lacrimal sac: a rare case of aggressive tumor and literature review. Med Pharm Rep.

[24] Carlin R, Henderson JW (1974). Malignant lymphoma of the nasolacrimal sac. Am J Ophthalmol.

[25] Jordan DR, Nerad JA (1988). Diffuse large-cell lymphoma of the nasolacrimal sac. Can J Ophthalmol.

[26] Venkitaraman R, George MK (2007). Primary non Hodgkin's lymphoma of the lacrimal sac. World J Surg Oncol.

[27] Kajita F, Oshitari T, Yotsukura J, Asanagi K, Baba T, Kishimoto T, Yamamoto S (2010). Case of primary diffuse large B-cell lymphoma of lacrimal sac in a Japanese patient. Clin Ophthalmol.

[28] Yahalom J, Illidge T, Specht L, Hoppe RT, Li YX, Tsang R, Wirth A (2015). Modern radiation therapy for extranodal lymphomas: field and dose guidelines from the International Lymphoma Radiation Oncology Group. Int J Radiat Oncol Biol Phys.

[29] Wirth A, Mikhaeel NG, Aleman BM (2020). Involved site radiation therapy in adult lymphomas: an overview of International Lymphoma Radiation Oncology Group Guidelines. Int J Radiat Oncol Biol Phys.

[30] Matsuo T, Ichimura K, Ichikawa T, Okumura Y, Kaji M, Yoshino T (2009). Positron emission tomography/computed tomography after immunocytochemical and clonal diagnosis of intraocular lymphoma with vitrectomy cell blocks. J Clin Exp Hematop.

[31] Matsuo T, Tanaka T (2019). Are there primary intraocular lymphomas that do not develop into central nervous system lymphomas?. J Clin Exp Hematop.

[32] Matsuo T, Yano T, Yoshio K, Tanaka T, Nishimura H, Matsuoka KI (2025). Whole-eye radiation for the local control of choroidal lymphoma in primary central nervous system lymphoma: a 14-year case study. Cureus.

[33] Matsuo T, Tanaka T, Ishida J, Kondo S, Matsuoka KI (2025). A natural course from primary intraocular lymphoma to brain lymphoma in four years according to patient's choice. Cureus.

[34] Fallico M, Chronopoulos A, Schutz JS, Reibaldi M (2021). Treatment of radiation maculopathy and radiation-induced macular edema: a systematic review. Surv Ophthalmol.

[35] Sahoo NK, Ranjan R, Tyagi M, Agrawal H, Reddy S (2021). Radiation retinopathy: detection and management strategies. Clin Ophthalmol.

